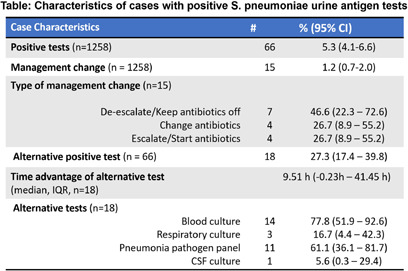# Is It Worth It? Assessing the Clinical Impact of the S. pneumoniae Urine Antigen Test

**DOI:** 10.1017/ash.2025.315

**Published:** 2025-09-24

**Authors:** Elizabeth Kim, Lucy Witt, Eli Wilber

**Affiliations:** 1 Emory University School of Medicine; 2 Emory University; 3 Division of Infectious Diseases, Department of Medicine, Emory University School of Medicine

## Abstract

**Background:** It is challenging to identify a pathogen in most cases of community acquired pneumonia (CAP) as most available diagnostic tests either lack sensitivity or require an invasive specimen. S. pneumoniae urine antigen test (SPUAT), which detects the most common cause of bacterial CAP, has been used due to its higher sensitivity, non-invasive specimen collection, and more rapid turnaround time. However, the most recent IDSA/ATS guidelines only weakly recommend obtaining SPUAT as results have limited effects on clinical management given current CAP treatment guidelines. Our study aimed to determine whether use of the SPUAT resulted in meaningful changes in clinical management within the Emory Healthcare system. **Method:** We studied all patients within our 6-hospital healthcare system who had a SPUAT performed between 12/1/2023 and 11/30/2024 (n = 1258). Chart review for each positive SPUAT case was performed by two separate reviewers to identify change in management based on SPUAT, alternative diagnostic tests that identified S. pneumoniae, and time to positivity of alternative diagnostic tests. Disagreements were adjudicated by discussion between the two reviewers. Proportions and 95% confidence intervals were calculated using prop.test in R version 4.3.1. **Result:** There were a total of 66 positive SPUAT out of 1258 total tests resulted (5.3%, 95%CI 4.1% – 6.6%) over 12 months. In 18 of the 66 positive SPUAT cases, an alternative diagnostic test was also positive for S. pneumoniae. In these cases, blood cultures were the most common alternative positive test (14/18) while the second most common alternative test was the pneumonia pathogen panel (11/18). In the majority (13/18) of cases with positive alternative tests, the alternative test resulted prior to the SPUAT. The median time to result for the first alternative test was 9.5 hours sooner than the SPUAT (IQR -0.2 hours - 37.9 hours). In 15 cases, a positive SPUAT resulted in a change in antibiotic management (1.2%, 95%CI 0.7%-2.0%). In cases where there was a change in management, de-escalation of antibiotics was the most common change in management identified (Table). The number of tests required for one management change was 84 tests at an estimated cumulative cost of $2100. **Conclusion:** In our healthcare system, SPUAT had a low test-positivity rate and an even lower rate of management changes per test ordered at a high cumulative cost per management change.

**Figure:**